# Long-Term Staff Acceptance of an AI-Supported Radar-Based Bed-Exit Surveillance System After Clinical Implementation

**DOI:** 10.3390/s26216720

**Published:** 2026-10-23

**Authors:** Valerie Götz, Tamara Eichenbrenner, Isabella Glaser, Vanessa Vater, Diana Fritsch, Sandra Noack, Manuela Pretto, Reto W. Kressig, Wolfgang Hasemann

**Affiliations:** 1 University Department of Geriatric Medicine Felix Platter, 4055 Basel, Switzerland; valerie.goetz1@icloud.com (V.G.); tamarae.eichenbrenner@t-online.de (T.E.); Isabella.Glaser@felixplatter.ch (I.G.); vanessavater103@gmail.com (V.V.); diana.fritsch87@gmail.com (D.F.); noack.sandra@ymail.com (S.N.); mpretto@bluewin.ch (M.P.); 2 Department of Clinical Research, Faculty of Medicine, University of Basel, 4031 Basel, Switzerland; reto-w.kressig@unibas.ch; 3 Institute of Nursing Science, Department of Public Health, University of Basel, 4056 Basel, Switzerland

**Keywords:** bed-exit surveillance, 3D-radar, infrared camera-based, artificial intelligence, fall prevention, delirium, geriatric nursing, health personnel, implementation evaluation, patient safety

## Abstract

**Highlights:**

**What are the main findings?**

- Staff acceptance of the radar-based bed-exit surveillance system remained high over three years of routine clinical use, although it was lower after initial technology selection and only partially increased by 2024.
- Long-term use revealed a differentiated acceptance pattern: mobile alerts and ease of use show higher acceptance, whereas false alarms and perceived burden showed lower acceptance rates.

**What are the implications of the main findings?**

- From the perspective of healthcare professionals, the reliable functioning and workflow fit of the two investigated bed-exit surveillance systems were more important for staff acceptance than the underlying sensing technology.
- Implementation strategies should prioritize reliable operation, few false alarms, low staff burden, and intuitive workflow integration, with acceptance reassessed during long-term routine use.

**Abstract:**

**Background:** Contactless bed-exit surveillance may support fall prevention, but little is known about healthcare professionals’ long-term acceptance after implementation. This study compared staff acceptance of two artificial intelligence-supported bed-exit surveillance technologies—an infrared camera-based system (Cogvis) and a three-dimensional radar-based system (QUMEA)—during technology selection and evaluated acceptance of the selected QUMEA system over three years of routine clinical use. **Methods:** Repeated cross-sectional surveys were conducted in 2021, 2022, and 2024 on a specialized acute geriatric ward for patients with delirium, yielding 52 questionnaires. Fourteen common acceptance items were rated on a four-point scale and dichotomized. Paired tests compared Cogvis and QUMEA in 2021, and mixed-effects logistic regression evaluated QUMEA acceptance over time. **Results:** QUMEA received higher positive ratings than Cogvis across nearly all items in 2021. The adjusted probability of a positive QUMEA response was 95.4% in 2021, 73.6% in 2022, and 86.4% in 2024 (overall *p* = 0.003). Acceptance remained lower in 2024 than in 2021 (*p* = 0.021). **Conclusions:** Staff acceptance remained high but was lower after technology selection and only partially reached former levels. Use over time revealed challenges related particularly to false alarms and perceived burden, emphasizing the importance of technical performance and workflow fit.

## 1. Introduction

Despite more than 40 years of development of bed-exit surveillance technologies, evidence regarding their effects on fall prevention, patient and staff outcomes, and technology acceptance remains limited. While the clinical outcomes of our QumPreFall study evaluating the contactless radar-based bed-exit surveillance system QUMEA (QUMEA AG, Solothurn, Switzerland) have been reported previously, this paper describes the technology selection process and presents previously unpublished data on staff acceptance of both the former and the current bed-exit surveillance systems [1].

In 1985, Widder described the Ambularm, a patient-attached mechanical bed-exit alarm connected to the bed or chair by a tether that generated an alarm when the patient attempted to leave the bed, thereby alerting nursing staff [2]. Evidence was initially limited to an uncontrolled survey demonstrating positive user perceptions but no proven effectiveness in fall prevention [3]. In 1988, Dubner et al. implemented an infrared bed-exit surveillance system and observed a significant 53% reduction in nighttime falls in a non-randomized before–after study, whereas daytime falls remained unchanged [4]. In 1993, Tideiksaar et al. evaluated a pressure-sensitive sensor pad positioned beneath the bed sheet that triggered an audible and visual alarm at the nursing station when pressure was released as the patient attempted to leave the bed. The study found no statistically significant difference in fall rates between the intervention and control groups. However, both groups received hourly visual checks, mechanical restraints, and routine use of half-length bed rails. False alarms were identified as the main barrier to staff acceptance, and in one out of 70 cases, the bed alarm was removed and replaced with mechanical restraints because of repeated false alarms [5].

In the following years, bed-exit surveillance technologies evolved considerably, including pressure-sensitive floor mats, mattress sensor pads, and tethered clip alarms [6]; dual-sensor systems combining pressure and infrared detection [7]; side rail-integrated sensor technology [8]; wearable accelerometer-based devices [9]; wireless modular bed-absence sensor systems [10]; and battery-less RFID-based wearable sensors [11]. More recent developments include continuous video monitoring [12], camera-assisted nursing observation [13], and contactless intelligent bed-exit information systems [14,15]. However, to our knowledge, no further published evidence exists about the outcomes of artificial intelligence (AI)-supported bed-exit surveillance in the context of optical sensors or cameras.

During the development of a specialised acute geriatric ward for patients with delirium (DelirUnit), bed rails were deliberately avoided, and low-floor beds were implemented instead to reduce injuries in patients at risk of unwanted bed-exits and falls. To further reduce the risk of injury, nurses placed a second mattress next to the low-floor bed when additional protection was required. During the development of the three-dimensional (3D) radar-based bed-exit surveillance system QUMEA, the commercially available infrared camera system Cogvis (Cogvis software und consulting GmbH, Vienna, Austria) was also evaluated. Despite technological advances, only a few studies have evaluated the effectiveness of bed-exit surveillance systems in preventing falls [4,5], whereas the acceptance of the inherent technology has been examined in only a limited number of studies [5,10,12,13,14,15]. Although previous studies have combined evaluations of clinical outcomes with staff experiences during implementation [15], no previous study has examined the clinical effectiveness of a bed-exit surveillance system together with healthcare professionals’ long-term acceptance following several years of routine clinical use. To our knowledge, no previous study has compared staff acceptance of two AI-supported contactless bed-exit surveillance systems within a real-world technology selection process and subsequently followed acceptance of the selected system over three years of routine clinical use. This study examines the long-term staff acceptance of a bed-exit surveillance system under real conditions in routine clinical care, complementing the clinical results of our QumPreFall study [1].

## 2. Materials and Methods

This study comprised two complementary analyses of staff acceptance of bed-exit surveillance systems on a specialised acute geriatric ward for patients with delirium (DelirUnit): (1) a comparison of staff acceptance of the commercially available AI-supported infra-red depth-sensing system Cogvis and the newly 3D radar-based system QUMEA during the 2021 technology selection process, and (2) a repeated cross-sectional evaluation of staff acceptance following implementation into routine clinical practice. Given the exploratory nature of the initial technology-selection survey and the absence of a preregistered analysis plan, no formal a priori hypotheses were specified. The study aimed to compare staff acceptance of the two systems during technology selection and to describe and evaluate changes in acceptance of the selected radar-based system during routine clinical use.

### 2.1. Study Design and Setting

This repeated cross-sectional survey was conducted on the DelirUnit of the University Department of Geriatric Medicine Felix Platter, Basel, Switzerland. The DelirUnit is a specialised acute geriatric ward providing interdisciplinary care for older patients with delirium. The concept is described in the Supplementary Materials and in our previous publication [1]. During the technology selection process in 2021, QUMEA was evaluated alongside Cogvis. Following implementation, QUMEA and the analogue CareMat^®^ contact mat placed in front of the bed were used in parallel throughout the clinical effectiveness study (2022–2023). After QUMEA had been selected, the system was further adapted in close collaboration between the manufacturer and the ward team: engineers regularly visited the unit, obtained feedback from staff on required functions and resolved initial technical problems. Throughout the study period, the manufacturer provided regular software updates, which did not change the functionality of the system but improved its detection accuracy. Staff members who joined the team later were trained using online training videos and were introduced to the system by experienced colleagues who had been involved since its implementation. The contact mat was discontinued in October 2023, after which QUMEA became the sole bed-exit surveillance system on the unit. The clinical effectiveness of QUMEA compared with CareMat^®^ regarding falls and nursing workload has been reported previously [1].

### 2.2. Participants

All members of the multidisciplinary DelirUnit team who routinely worked with the bed-exit surveillance systems were invited to participate. Eligible participants included registered nurses, nursing assistants, physicians, physiotherapists, occupational therapists, chaplain, and other healthcare professionals involved in direct patient care. Temporary staff members and employees without regular experience using the respective surveillance system were not eligible to participate. Participation was voluntary, and all questionnaires were completed anonymously.

### 2.3. Survey Instrument

Staff acceptance was assessed using a self-developed structured questionnaire originally designed for the 2021 technology selection process. The questionnaire originally comprised 20 acceptance items. Fourteen corresponding acceptance items were included in all QUMEA surveys (2021, 2022, and 2024) and constituted the primary outcome measure for assessing staff acceptance across survey years (Tables S1–S4). One additional system-specific item differed between the Cogvis and QUMEA questionnaires. Responses were recorded on a four-point Likert scale ranging from very negative to very positive. Participants were also invited to provide free-text comments. In 2024, the questionnaire was expanded to include 42 closed- and open-ended questions while retaining the original acceptance items (Table S4). Minor wording refinements were introduced in the 2024 questionnaire to improve clarity and reduce ambiguity. These refinements did not alter the underlying content, and the corresponding items were therefore treated as equivalent across survey years.

### 2.4. Data Collection

The initial technology selection survey was conducted in 2021 using paper-based questionnaires. Follow-up surveys were conducted in 2022 using Inquery^®^ Survey Server (Inworks GmbH, Berlin, Germany) and in 2024 using REDCap (Vanderbilt University Medical Center, Nashville, TN, USA). Eligible staff members received written information about the study before completing the anonymous questionnaires. No direct personal identifiers were collected.

### 2.5. Statistical Analysis

Questionnaire data were analysed using R version 4.6.1 (R Foundation for Statistical Computing, Vienna, Austria). Descriptive results are presented as frequencies and percentages. Questionnaire responses were dichotomised into positive (+1/+2) and negative (−2/−1) categories. Responses outside these four categories, including ‘Cannot be assessed,’ were treated as missing.

For the 2021 technology selection process, analyses were restricted to participants who completed evaluations of both surveillance systems. Paired item-level comparisons between Cogvis and QUMEA were performed using exact McNemar tests.

For the repeated cross-sectional QUMEA surveys (2021, 2022, and 2024), analyses were restricted to the 14 questionnaire items common to all survey years. Staff acceptance was analysed using a mixed-effects logistic regression model including survey year and professional group as fixed effects and random intercepts for participant and questionnaire item. The overall effect of survey year was assessed using a likelihood-ratio test comparing models with and without survey year. Adjusted probabilities and 95% confidence intervals were estimated using estimated marginal means. Three planned contrasts were evaluated without multiplicity adjustment: the primary comparison between 2021 and 2024, the secondary comparison between 2021 and 2022, and the exploratory comparison between 2022 and 2024. Three questionnaires from 2022 without information on professional group were excluded from the adjusted analyses, which were therefore based on 49 questionnaires.

Each questionnaire item was analysed separately using bias-reduced logistic regression including survey year and professional group as fixed effects. Adjusted probabilities and 95% confidence intervals were estimated using estimated marginal means. The overall effect of survey year within each item was evaluated using joint Wald tests.

Because questionnaires were completed anonymously, repeated participation of individual healthcare professionals across survey years could not be identified. Consequently, the three survey waves were analysed as independent repeated cross-sectional samples. 

### 2.6. Ethical Considerations

The survey was conducted as an anonymous evaluation of healthcare professionals and did not involve patient data. The main study using patient data was registered on ClinicalTrials.gov (NCT05391334) and was approved by the Ethics Committee of Northwestern and Central Switzerland (EKNZ) (BASEC No. 2021-02501). According to the EKNZ, the survey did not fall within the scope of the Swiss Human Research Act and therefore did not require additional formal ethical approval.

## 3. Results

Across the three survey waves, 52 questionnaires were completed (Table 1).

### 3.1. Results of the Technology Selection Process

**Figure 1:**
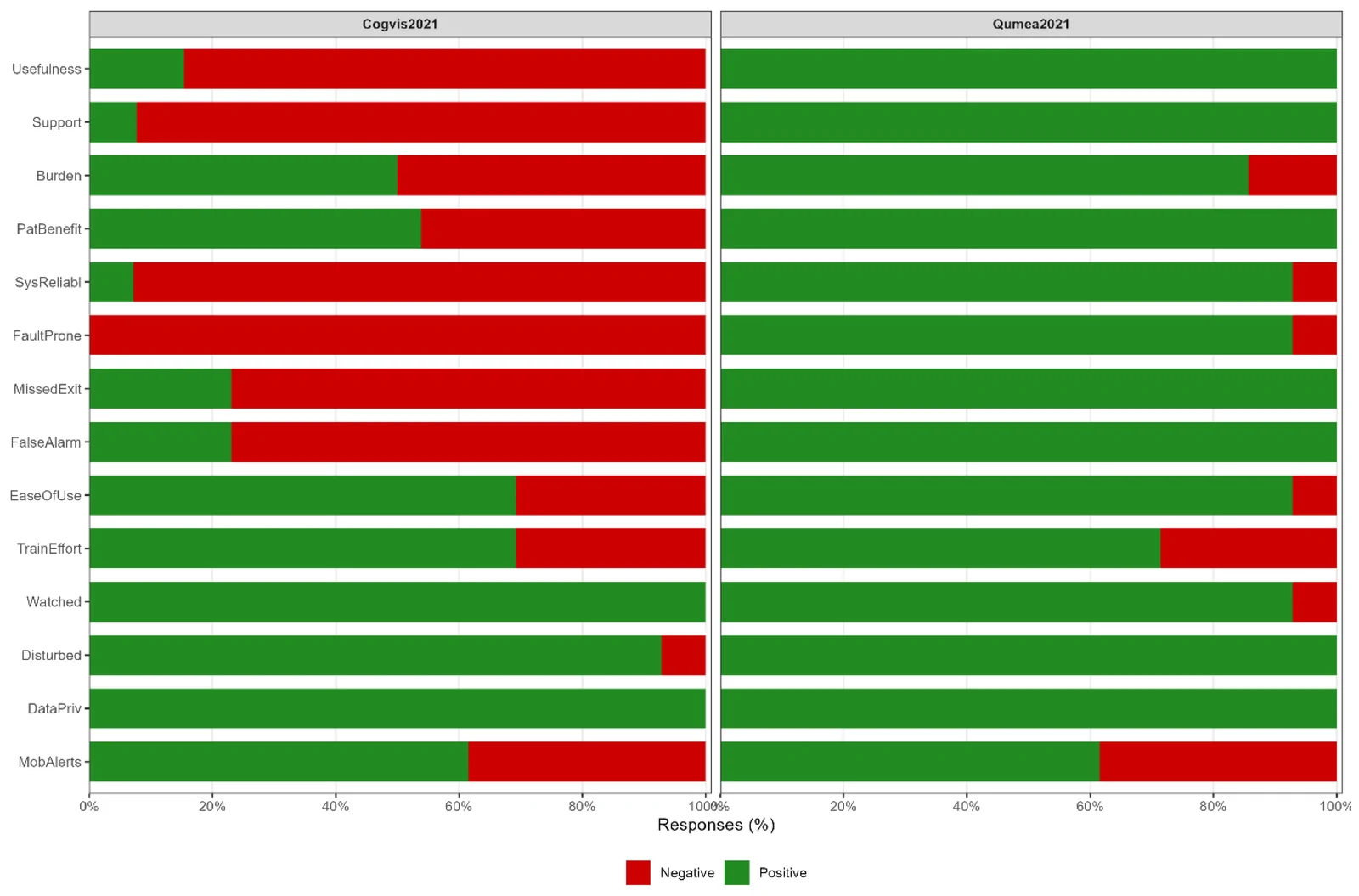
Positive and negative responses by questionnaire item: Cogvis and QUMEA (2021). Legend: Percentage of positive and negative responses to the individual questionnaire items for AI-supported contactless infra-red bed-exit surveillance Cogvis (2021) and AI-supported contactless 3D-radar bed-exit surveillance QUMEA (2021). Responses were dichotomised into positive (+1/+2) and negative (−2/−1). Item labels and their corresponding questionnaire wording are provided in Table S5.

In 2021, *14* healthcare professionals evaluated both Cogvis and QUMEA, enabling paired comparisons of staff acceptance between the two systems. Across nearly all questionnaire items, QUMEA received a markedly higher proportion of positive responses than Cogvis (Figure 1). The largest differences were observed for perceived Usefulness, Support, System Reliability, Prone to Faults, Missed Bed-exit, and False Alarms, whereas positive ratings for Data Privacy were high for both systems (Figure 1). Based on 13 or 14 valid pairs per item, the unadjusted exact McNemar *p* values were <0.001 for Usefulness, Support, System Reliability, and Prone to Faults; 0.002 for Missed Bed-exit and False Alarms; and 0.031 for Patient Benefit.

### 3.2. Staff Acceptance During Routine Clinical Use

**Figure 2:**
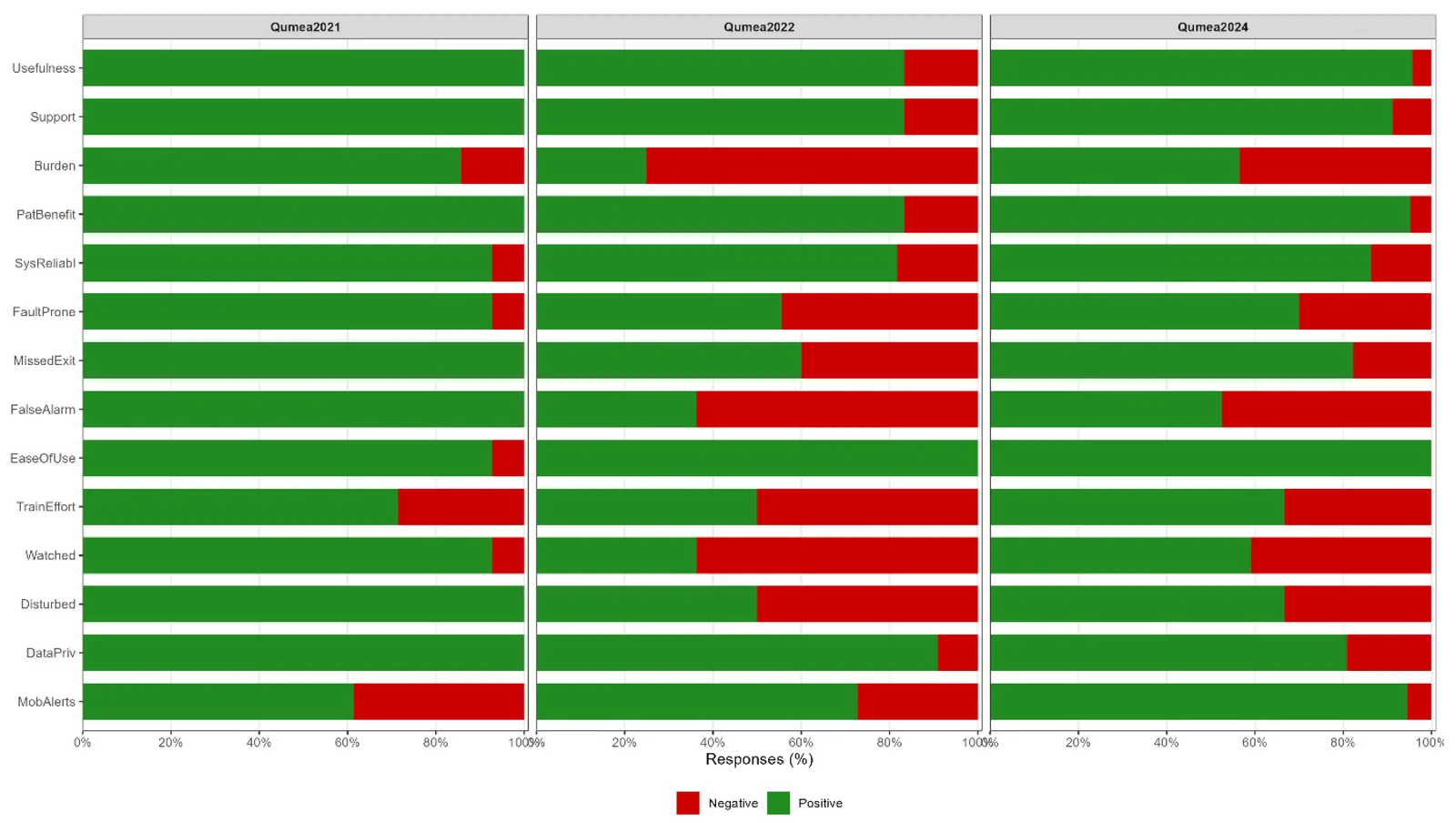
Positive and negative responses across the 14 common questionnaire items by survey year. Legend: Percentages of valid responses to the 14 questionnaire items included in all QUMEA surveys conducted in 2021, 2022, and 2024. Responses were dichotomised as positive (+1/+2) or negative (−2/−1). Item labels and their corresponding questionnaire wording are provided in Table S5.

Overall, positive responses predominated across the 14 questionnaire items in each survey year (Figure 2). Compared with 2021, a higher proportion of negative responses was observed across several questionnaire items in 2022. In 2024, positive responses increased again for most items, although the high level of acceptance observed in 2021 was not consistently regained.

The descriptive findings were confirmed by the mixed-effects logistic regression model (Table 2). Staff acceptance differed significantly across survey years (likelihood-ratio χ^2^ = 11.36, df = 2, *p* = 0.003). The adjusted probability of a positive response was 95.4% (95% CI 88.0–98.3) in 2021, decreased to 73.6% (95% CI 50.8–88.2) in 2022, and increased to 86.4% (95% CI 72.6–93.8) in 2024.

A sensitivity analysis using the original four-point responses in a cumulative link mixed model confirmed the overall survey-year effect (*p* = 0.019) and the lower acceptance in 2022 compared with 2021; the lower acceptance in 2024 compared with 2021 was consistent in direction but less robust (Table S6).

The odds of a positive response were significantly higher in 2021 than in 2024 (OR 3.27, 95% CI 1.19–8.96; *p* = 0.021). The odds of a positive response were also significantly higher in 2021 than in 2022 (OR 7.43, 95% CI 2.37–23.27; *p* < 0.001). Although the odds of a positive response were higher in 2024 than in 2022, the difference was not statistically significant (2022 vs. 2024: OR 0.44, 95% CI 0.18–1.08; *p* = 0.072). 

The item-level analyses showed differing patterns in the development of individual aspects of staff acceptance across survey years (Table 3). Positive staff acceptance increased between 2021 and 2024 for Mobile alerts (+33.1 percentage points) and Ease of use (+14.4 percentage points), while Training effort remained essentially unchanged (+0.7 percentage points). All remaining questionnaire items showed lower adjusted probabilities of positive responses in 2024 than in 2021. The largest change was observed for False Alarms (−42.1 percentage points), followed by Disturbed (−29.5 percentage points), Burden (−28.3 percentage points), Watched (−27.6 percentage points), and Prone to Faults (−23.1 percentage points) (Table 3).

After Holm adjustment for multiple comparisons across the 14 items, none of the item-level changes across survey years was statistically significant; the item-level results are therefore presented as descriptive patterns.

## 4. Discussion

To our knowledge, this is the first study to compare healthcare professionals’ acceptance of an AI-supported contactless 3D radar-based bed-exit surveillance system (QUMEA) with an AI-supported contactless infrared camera-based bed-exit surveillance system (Cogvis) and to show a clear preference for the radar-based technology. Previous studies have predominantly evaluated the implementation, usability, or effectiveness of individual bed-exit surveillance systems. In contrast, our study examined healthcare professionals’ preferences during a direct technology selection process between two competing AI-supported systems. The findings suggest that staff preference was primarily driven by the observed technical performance of the systems in everyday clinical use rather than by the underlying AI technology itself.

Staff acceptance remained high throughout the observation period but was significantly lower following implementation into routine clinical practice. Although acceptance reached a temporary low point during the COVID-19 pandemic in 2022, it partially increased in 2024 without returning to the exceptionally high level observed in 2021.

Item-level analyses demonstrated that the long-term development of staff acceptance was not uniform across questionnaire items. While acceptance improved or remained stable for only three aspects (Mobile Alerts, Ease of Use, and Training Effort), all remaining questionnaire items showed lower adjusted probabilities of positive responses in 2024 than in 2021, with the largest changes observed for False Alarms, Disturbed, Burden, Watched, and Prone to Faults.

An important finding of the present study is that staff preference during the 2021 technology selection process was primarily determined by technical performance rather than by the underlying AI technology itself. Healthcare professionals preferred the system that functioned more reliably under routine clinical conditions and better supported their workflow. The preference for QUMEA was driven by technical limitations of the former Cogvis system: Cogvis was unable to reliably detect bed-exits when low-floor beds were used in their lowest position, detection was impaired when patient rooms were exposed to bright sunlight, and bed-exit zones had to be manually configured via a web-based interface, which proved impractical for bedside nursing staff. None of these limitations were observed with QUMEA. Our findings are broadly in line with observations of Walzer et al., who reported that nurses’ perspectives are strongly influenced by the technical performance of bed-exit information systems. Their mixed-methods study demonstrated that successful implementation depends on aligning nurses’ expectations with actual system performance and highlighted information overload, alarm fatigue, and technical specifications as major determinants of perceived usefulness [15].

One plausible explanation for the temporary lower staff acceptance observed in 2022 is the extraordinary working conditions during the COVID-19 pandemic. To protect non-infected patients, isolation areas had to be established on the DelirUnit. Patients with delirium frequently did not understand or adhere to isolation measures. In accordance with the unit’s philosophy of avoiding mechanical and pharmacological restraints, patients remained free to mobilise within their rooms. Consequently, healthcare professionals had to respond repeatedly to bed-exit warnings while simultaneously donning and removing personal protective equipment when entering and leaving isolation rooms. These conditions are likely to have increased staff workload and may have contributed to the temporary lower staff acceptance observed during this period. An increase in workload associated with bed-exit surveillance was already reported by Tideiksaar et al. in 1993, where repeated false alarms led to increased use of mechanical restraints and removal of the bed-exit surveillance system [5]. However, workload was not assessed in the survey, and this explanation therefore remains a hypothesis.

Despite a more critical evaluation of several individual acceptance aspects, staff acceptance remained high throughout the study. This suggests that healthcare professionals distinguished between limitations of specific system characteristics and the overall clinical value of the technology. In routine practice, the perceived benefits for patient safety and clinical workflow appear to outweigh the disadvantages associated with individual system features. In contrast to our findings, Laramee et al. reported that nurses’ attitudes towards an electronic health record became progressively less positive during the first 18 months after implementation. The authors attributed this difference primarily to increased documentation burden, redundant documentation and workflow inefficiencies [16].

One encouraging finding of the present study is that several implementation-related aspects of staff acceptance improved or remained stable during long-term routine use. Positive ratings increased for Mobile Alerts (+33.1 percentage points) and Ease of Use (+14.4 percentage points), whereas Training Effort remained essentially unchanged (+0.7 percentage points). These findings suggest that the practical integration of QUMEA into everyday clinical practice improved over time. Although the temporal pattern differed from our observations, Zadvinskis et al. [17] likewise demonstrated that nurses’ perceptions of newly implemented health information technology were not static but evolved over time, with dissatisfaction peaking approximately 9 months after implementation before becoming more balanced after 18 months.

As healthcare professionals gained experience with the system, its operation was perceived as increasingly intuitive, while the effort required to train new staff remained stable. The marked improvement in the acceptance of mobile alerts further indicates that mobile notification became increasingly integrated into routine clinical workflows.

A second important finding is the stability of several acceptance items following long-term implementation. Positive ratings for Patient Benefit, System Reliability, Usefulness, and Support changed only marginally between the technology selection process in 2021 and the follow-up survey in 2024, with absolute differences ranging from 2.0 to 8.2 percentage points. These findings indicate that the favourable initial evaluation of QUMEA was largely maintained throughout routine clinical use. Healthcare professionals continued to perceive the system as clinically useful, supportive of their work, beneficial for patients, and technically reliable, suggesting that the system’s perceived value extended well beyond the initial technology selection process.

In contrast, several aspects of routine clinical use were evaluated less favourably after long-term implementation than during the initial technology selection process. The largest differences were observed for False Alarms, Disturbed, Burden, Watched, Prone to Faults, Data Privacy, and Missed Bed-exit. Rather than representing isolated findings, these items describe a common pattern. During the technology selection process, staff primarily evaluated the expected benefits of a new surveillance system under relatively controlled conditions. Following several years of routine clinical use, however, healthcare professionals had accumulated extensive practical experience with the technology, resulting in a more differentiated evaluation that increasingly reflected the challenges and limitations encountered in everyday clinical practice. Importantly, this more critical appraisal of individual acceptance aspects was not accompanied by poorer clinical performance. On the contrary, the accompanying QumPreFall study demonstrated that routine implementation of QUMEA was associated with a clinically relevant 49% reduction in bed-exit-related falls, 20% fewer bed-exit warnings, and a 30% reduction in nurse presence time compared with the analogue CareMat [1]. These findings suggest that long-term user experience leads to a more balanced and critical evaluation of individual system characteristics without diminishing the perceived overall clinical value of the technology.

The present findings suggest that future development of AI-supported bed-exit surveillance systems should focus not only on basic functionality but increasingly on reducing false alarms, improving workflow integration and enhancing user experience during long-term routine use.

This study has several limitations. First, it was conducted in a single specialised delirium unit, which may limit the generalisability of the findings to other clinical settings. Second, staff acceptance was evaluated using repeated cross-sectional surveys with modest sample sizes. Because questionnaires were completed anonymously, individual participants could not be followed across survey years, precluding the analysis of within-person changes over time. In addition, the questionnaire was developed specifically for the technology selection process and was not formally validated. Third, staff acceptance represents a subjective evaluation of the technology and may not necessarily reflect its objective clinical performance. However, the acceptance data can be interpreted alongside the previously published clinical outcomes of the QumPreFall study, which demonstrated clinically relevant reductions in bed-exit-related falls, bed-exit warnings, and nurse presence time following implementation of QUMEA. Although the number of positions on the unit remained constant throughout the study period, individual position holders changed over time. Because invitations were distributed via an internal e-mail distribution list whose historical membership could not be retrieved, the number of invited staff members and the response rates could not be determined. Differences between survey years may therefore partly reflect differences in participation and staff composition. 

In 2021, Cogvis and QUMEA were operated in parallel in routine clinical practice, and staff assessed QUMEA in direct comparison with Cogvis during the technology selection process. In 2022 and 2024, QUMEA was the sole bed-exit surveillance system on the unit at the time of the surveys. The higher acceptance observed in 2021 may therefore partly reflect this comparative selection context rather than a change over time alone. 

The comparison of Cogvis and QUMEA in 2021 reflects staff acceptance of the two systems as experienced in routine clinical practice during the technology selection process. Cogvis provided the same functionality as the previously used contact mats and alerted staff via the nurse call system, whereas QUMEA was operated as a stand-alone system. Because exposure, training, system configuration and integration into the ward infrastructure were not standardised, the results should not be interpreted as a technical comparison of the systems or their underlying sensing technologies. 

The sample was small and reflects the size and interprofessional composition of a single hospital ward team, resulting in wide confidence intervals. However, sensitivity analyses yielded consistent results (Table S6).

At present, new bed-exit surveillance systems are frequently introduced to the market without evidence of their clinical effectiveness or their acceptance by healthcare professionals and patients in routine care. Future evaluations should therefore assess these dimensions systematically, before market introduction and procurement as well as at regular intervals during routine use, and in different clinical settings and patient populations. In addition to staff acceptance, the acceptance by patients and, particularly in patients with cognitive impairment, by their relatives should be considered.

## 5. Conclusions

The findings suggest that the long-term success of AI-supported bed-exit surveillance depends not only on clinical effectiveness, but also on addressing implementation barriers identified by healthcare professionals, particularly perceived burden, false alarms, system reliability and concerns related to feelings of being watched and disturbed in nursing processes. Staff acceptance and clinical effectiveness may therefore be regarded as complementary dimensions of successful implementation.

For healthcare professionals, the underlying sensing technology of the two investigated systems was less important than whether the system functioned reliably in daily practice, alerting staff to bed-exits without producing too many or too few alarms. From a practical perspective, reduction in staff burden, reliability, few false alarms and missed events, and ease of use may therefore be important considerations when selecting and implementing bed-exit surveillance systems. Because these characteristics may be assessed differently after prolonged routine use than during initial testing, staff acceptance and system performance should be evaluated both before implementation and at regular intervals thereafter.

## Figures and Tables

**Table 1:** Survey participants.

Professional Group	2021	2022	2024
Registered nurses (incl. students)	7	5	10
Healthcare assistants (incl. students)	2	2	3
Nursing assistants (incl. students)	2	5	8
Physicians/Therapists/Other healthcare professionals	3	0	2
Undisclosed profession	0	3	0
Total participants	14	15	23

**Table 2:** Staff acceptance of QUMEA across survey years.

Adjusted Probabilities			
Survey Year	Adjusted Probability of a Positive Response % (95% CI)		
2021	95.4 (88.0–98.3)		
2022	73.6 (50.8–88.2)		
2024	86.4 (72.6–93.8)		
Overall survey-year effect			
Effect	**Likelihood-ratio** **chi-square**	**df**	***p***
Survey year	11.36	2	0.003
Planned contrasts			
Planned contrast	**Odds ratio** **(95% CI)**	***p***	
Primary: 2021 vs. 2024	3.27 (1.19–8.96)	0.021	
Secondary: 2021 vs. 2022	7.43 (2.37–23.27)	<0.001	
**Exploratory: 2022 vs. 2024**	0.44 (0.18–1.08)	0.072	

Values are adjusted probabilities and 95% confidence intervals estimated from a mixed-effects logistic regression model including survey year and professional group as fixed effects and random intercepts for participant and questionnaire item. The overall survey-year effect was assessed using a likelihood-ratio test. Planned contrasts were evaluated without multiplicity adjustment. Positive responses were defined as ratings of +1 or +2; negative responses as −2 or −1.

**Table 3:** Adjusted staff acceptance by questionnaire item across survey years.

Rank (Change 2021–2024)	Acceptance Aspect	2021 % (95% CI)	2022 % (95% CI)	2024 % (95% CI)	Change 2021–2024 Percentage Points	Global Year *p*
1	MobAlerts	55.1 (26.8–80.4)	62.1 (26.1–88.4)	88.2 (59.2–97.5)	33.1	0.200
2	EaseOfUse	83.4 (50.5–96.1)	97.2 (60.6–99.9)	97.8 (72.2–99.9)	14.4	0.329
3	TrainEffort	64.6 (35.0–86.1)	48.7 (20.0–78.4)	65.3 (40.2–84.0)	0.7	0.673
4	PatBenefit	94.4 (59.5–99.5)	80.8 (39.2–96.5)	92.4 (65.3–98.7)	−2	0.554
5	SysReliabl	88.8 (58.3–97.8)	84.0 (46.6–96.9)	86.1 (60.3–96.2)	−2.7	0.938
6	Usefulness	94.6 (61.2–99.5)	78.2 (39.4–95.2)	91.9 (68.4–98.3)	−2.7	0.439
7	Support	94.0 (56.7–99.5)	77.5 (39.3–94.8)	85.9 (61.2–95.9)	−8.2	0.588
8	MissedExit	95.0 (63.4–99.5)	56.4 (17.7–88.6)	80.4 (40.6–96.1)	−14.6	0.183
9	DataPriv	94.7 (58.8–99.6)	88.6 (50.0–98.4)	76.9 (49.4–91.9)	−17.8	0.411
10	FaultProne	86.6 (54.4–97.2)	56.9 (21.0–86.7)	63.5 (37.6–83.4)	−23.1	0.358
11	Watched	88.7 (56.5–97.9)	43.8 (14.0–78.9)	61.1 (33.4–83.1)	−27.6	0.109
12	Burden	81.1 (51.2–94.6)	24.2 (7.4–56.1)	52.8 (30.4–74.1)	−28.3	0.037
13	Disturbed	96.1 (59.8–99.8)	57.6 (22.5–86.4)	66.6 (37.8–86.7)	−29.5	0.171
14	FalseAlarm	95.5 (60.6–99.7)	42.9 (13.0–79.1)	53.5 (23.9–80.7)	−42.1	0.072

Adjusted probabilities and 95% confidence intervals were estimated using separate bias-reduced logistic regression models for each item, including survey year and professional group. Items are ranked according to the change in adjusted positive responses between 2021 and 2024, from the largest increase to the largest decrease. The global year *p* value is based on a joint two-degree-of-freedom Wald test within each item model. Item labels and their corresponding questionnaire wording are provided in Table S5.

## Data Availability

The data underlying this article are not publicly available because the small study population could permit the identification of individual participants. The R code is not publicly available because it consists of project-specific analysis scripts that have not been prepared for external distribution. The study materials are included in the Supplementary Materials.

## Supplementary Materials

The following supporting information can be downloaded at https://www.mdpi.com/article/10.3390/s26216720/s1, Table S1. Cogvis 2021. Coding of the Cogvis 2021 infrared bed-exit surveillance questionnaire; Table S2. QUMEA 2021. Coding of the QUMEA 2021 3D-radar bed-exit surveillance questionnaire; Table S3. QUMEA 2022. Coding of Questionnaire 3D-radar bed-exit surveillance QUMEA 2022; Table S4. QUMEA 2024. Coding of Questionnaire 3D radar bed-exit surveillance QUMEA 2024; Table S5. Mapping of publication labels, internal variable names and questionnaire items. Table S6: Sensitivity analyses of staff acceptance of QUMEA across survey years.
