# Supplementary material for: Long-Term Staff Acceptance of an AI-Supported Radar-Based Bed-Exit Surveillance System After Clinical Implementation
Source: Sensors (Basel). 2026 Oct 23;26(21):6720. doi: 10.3390/s26216720 (PMC13765659; doi:10.3390/s26216720)
Supplement: Supplementary file 1 [file sensors-26-06720-s001.zip › sensors-4601815-supplementary.pdf]

Supplementary Material

Table S1. Cogvis 2021

Coding of the Cogvis 2021 infrared bed-exit surveillance questionnaire

Benefits of Cogvis 2021

(1) How would you rate the overall benefits of Cogvis? [a)]
(2) Do you feel that Cogvis provides targeted support for your work? [b)]
(3) Does Cogvis place an additional burden on you? [c)]
(4) Do you think that Cogvis benefits patients? [b)]

Performance of Cogvis 2021

(5) How reliable do you consider Cogvis to be? [f)]
(6) How prone to faults was Cogvis? [g)]
(7) How often did undetected bed exits occur? [g)]
(8) How often did false alarms occur? [g)]

Ease of use of Cogvis 2021

(9) How easy is Cogvis to use? [d)]
(10) How much effort is involved in explaining Cogvis to a new member of staff? [c)]

Privacy & security Cogvis 2021

(11) Do you feel as though Cogvis is watching you? [c)]
(12) Do you feel disturbed by Cogvis? [c)]
(13) Do you have any concerns about data protection with Cogvis? [c)]

Cogvis alerts 2021

(14) Do you prefer to receive alerts on your mobile phone? [b)]
(15) Do you prefer to receive alerts via the paging system? [d)]

Alternatives to Cogvis 2021

(16) Would you prefer to use contact mats instead of Cogvis? [c)]
(17) Would you prefer to use Cognimat mattresses instead of Cogvis? [c)]
(18) Would you advocate purchasing Cogvis? [b)]
(19) Would it make sense to use Cogvis in a 'normal' ward? [b)]

Cogvis 2021 Service

(20) Hotline availability [e)]
(21) Cooperation with the company [e)]

a) (2) High (1) Rather high (-1) Somewhat low (-2) Low () Cannot be assessed
b) (2) Applies (1) Somewhat true (-1) Not really true (-2) Does not apply () Cannot be assessed
c) (2) Does not apply (1) Not really true (-1) Somewhat applies (-2) Applies () Cannot be assessed
d) (2) Very easy (1) easy (-1) Difficult (-2) very difficult () Cannot be assessed
e) (2) Very good (1) Good (-1) rather poor (-2) very poor () Cannot be assessed
f) (2) to the highest degree (1) to a high degree (-1) to a small extent (-2) to a very small extent () Cannot be assessed
g) (2) never (1) rarely (-1) often (-2) always () Cannot be assessed

© Wolfgang Hasemann

Table S2. QUMEA 2021

Coding of the QUMEA 2021 3D-radar bed-exit surveillance questionnaire

Benefits of QUMEA 2021

(1) How would you rate the overall benefits of QUMEA? [a)]
(2) Do you feel that QUMEA provides targeted support for your work? [b)]
(3) Does QUMEA place an additional burden on you? [c)]
(4) Do you think that QUMEA benefits patients? [b)]

Performance of QUMEA 2021

(5) How reliable do you consider QUMEA to be? [f)]
(6) How prone to faults was QUMEA? [g)]
(7) How often did undetected bed exits occur? [g)]
(8) How often did false alarms occur? [g)]

Ease of use of QUMEA 2021

(9) How easy is QUMEA to use? [d)]
(10) How much effort is involved in explaining QUMEA to a new member of staff? [c)]

Privacy & security QUMEA 2021

(11) Do you feel that QUMEA is watching you? [c)]
(12) Do you feel that QUMEA is intrusive? [c)]
(13) Do you have any concerns about data protection with QUMEA? [c)]

QUMEA alerts 2021

(14) Do you prefer to receive alerts on your mobile phone? [b)]
(15) Do you prefer alerts on the intercom system? [d)]

QUMEA alternatives 2021

(16) Would you prefer to use contact mats instead of QUMEA? [c)]
(17) Would you prefer to use Cognimat mattresses instead of QUMEA? [c)]
(18) Would you advocate purchasing QUMEA?
(19) Would it make sense to use QUMEA in a 'normal' ward? [b)]

QUMEA Service 2021

(20) Hotline availability [e)]
(21) Cooperation with the company [e)]

a) (2) High (1) Somewhat high (-1) Somewhat low (-2) Low () Cannot be assessed
b) (2) Applies (1) Somewhat true (-1) Not really true (-2) Does not apply () Cannot be assessed
c) (2) Does not apply (1) Does not really apply (-1) Somewhat applies (-2) Applies () Cannot be assessed
d) (2) Very easy (1) easy (-1) Difficult (-2) very difficult () Cannot be assessed
e) (2) Very good (1) good (-1) rather poor (-2) very poor () Cannot be assessed
f) (2) to the highest degree (1) to a high degree (-1) to a small extent (-2) to a very small extent () Cannot be assessed
g) (2) never (1) rarely (-1) often (-2) always () Cannot be assessed

© Wolfgang Hasemann

Table S3. QUMEA 2022

Coding of Questionnaire 3D-radar bed-exit surveillance QUMEA 2022

Benefits of QUMEA 2021

(1) How would you rate the overall benefits of QUMEA? [a)]
(2) Do you feel that QUMEA provides targeted support for your work? [b)]
(3) Does QUMEA place an additional burden on you? [c)]
(4) Do you think that QUMEA benefits patients? [b)]

Performance of QUMEA 2021

(5) How reliable do you consider QUMEA to be? [f)]
(6) How prone to faults was QUMEA? [g)]
(7) How often did undetected bed exits occur? [g)]
(8) How often did false alarms occur? [g)]

Ease of use of QUMEA 2021

(9) How easy is QUMEA to use? [d)]
(10) How much effort is involved in explaining QUMEA to a new member of staff? [c)]

Privacy & security QUMEA 2021

(11) Do you feel as though QUMEA is watching you? [c)]
(12) Do you feel that QUMEA is intrusive? [c)]
(13) Do you have any concerns about data protection with QUMEA? [c)]

QUMEA alerts 2021

(14) Do you prefer to receive alerts on your mobile phone? [b)]
(15) Do you prefer alerts on the intercom system? [d)]

QUMEA alternatives 2021

(16) Would you prefer to use contact mats instead of QUMEA? [c)]
(17) Would you advocate for the purchase of QUMEA? [b)]
(18) Would it make sense to use QUMEA in a 'normal' ward? [b)]

QUMEA service 2021

(19) Hotline availability [e)]
(20) Cooperation with the company [e)]

a) (2) Large (1) Rather large (-1) Somewhat low (-2) Low () Cannot be assessed
b) (2) Applies (1) Somewhat true (-1) Not really true (-2) Does not apply () Cannot be assessed
c) (2) Does not apply (1) Not really true (-1) Somewhat applies (-2) Applies () Cannot be assessed
d) (2) Very easy (1) easy (-1) Difficult (-2) very difficult () Cannot be assessed
e) (2) Very good (1) good (-1) rather poor (-2) very poor () Cannot be assessed
f) (2) to the highest degree (1) to a high degree (-1) to a small extent (-2) to a very small extent () Cannot be assessed
g) (2) never (1) rarely (-1) often (-2) always () Cannot be assessed

© Wolfgang Hasemann

Table S4. QUMEA 2024

Coding of Questionnaire 3D radar bed-exit surveillance QUMEA 2024
**General QUMEA 2024**<br>(3) My overall assessment of QUMEA is [a)]<br>(4) QUMEA fulfils its purpose. [b)]<br>(5) QUMEA causes me stress. [c)]<br>**Benefits of QUMEA 2024**<br>(6) How would you rate the overall benefits of QUMEA? [a)]<br>(7) Do you feel that QUMEA provides targeted support for your work? [b)]<br>(8) Does QUMEA place an additional burden on you? [c)]<br>(9) Do you think that QUMEA benefits patients? [b)]<br>(10) I can tailor QUMEA effectively to patients' needs. [b)]<br>(11) QUMEA gives me greater confidence in my work. [b)]<br>**Performance of QUMEA 2024**<br>(13) How reliable do you rate QUMEA? [f)]<br>(14) How prone to faults is QUMEA? [g)]<br>(15) How often did QUMEA fail to issue a warning even though a patient had got out of bed? [g)]<br>(16) How often did false alarms occur? [g)]<br>(17) How often did QUMEA fail to trigger an alarm even though a fall occurred? [g)]<br>(18) How often did false alarms occur? [g)]<br>(19) I can rely on QUMEA. [b)]<br>**Ease of use of QUMEA 2024**<br>(20) How easy is QUMEA to use? [d)]<br>(21) How much effort does it take to explain QUMEA to a new employee? [h)]<br>(22) How much effort does it take to explain QUMEA to a pool employee? [h)]<br>(23) How much effort does it take to explain QUMEA to a temporary employee (from an external company)? [h)]<br>(24) I quickly got to grips with how to use QUMEA. [b)]<br>(25) The app is easy to use. [b)]<br>(26) Can any queries regarding the use of QUMEA be resolved promptly? [b)]<br>**Privacy & security QUMEA 2024**<br>(27) Do you feel that QUMEA is monitoring you? [c)]<br>(28) Do you feel that QUMEA is intrusive? [c)]<br>(29) Do you have any concerns about data protection with QUMEA? [c)]<br>(30) Do patients feel monitored by QUMEA? [c)]<br>(31) Do patients or their relatives have concerns regarding privacy? [c)]<br>**QUMEA Alerts 2024**<br>(32) Do you prefer to receive warnings/alerts on your mobile phone? [b)]<br>(33) Do you prefer to receive alerts/alarms via the call system? [c)]<br>(34) Is it easy to tell the difference between the alerts/alarms from the call system and those from QUMEA? [b)]<br>(35) I find the alerts disruptive. [c)]<br>(36) I can respond quickly to the alerts. [b)]<br>(37) What priority does a 'get up' alert have in your work? [i)]<br>(38) What priority does a fall alarm have in your work? [i)]
**a)** (2) High (1) Quite high (-1) Somewhat low (-2) Low () Cannot be assessed<br>**b)** (2) Applies (1) Somewhat true (-1) Not really true (-2) Does not apply () Cannot be assessed<br>**c)** (2) Does not apply (1) Not really true (-1) Somewhat applies (-2) Applies () Cannot be assessed<br>**d)** (2) Very easy (1) easy (-1) Difficult (-2) very difficult () Cannot be assessed<br>**e)** (2) Very good (1) good (-1) rather poor (-2) very poor () Cannot be assessed<br>**f)** (2) to the highest degree (1) to a high degree (-1) to a small extent (-2) to a very small extent () Cannot be assessed

**g)** (2) never	(1) rarely	(-1) often	(-2) always	() Cannot be assessed
**h)** (2) very low	(1) low	(-1) high	(-2) very high	() Cannot be assessed
**i)** (-2) very low	(-1) low	(1) high	(2) very high	() Cannot be assessed

© Wolfgang Hasemann

Table S5. Mapping of publication labels, internal variable names and questionnaire items

Item-ID	Publication label	Label in text	Internal variable	German variable	Reference questionnaire item (English)	Original questionnaire item (German)
1	Usefulness	Usefulness	Useful	nutzen	How would you rate the overall benefits of Cogvis?	Wie schätzt du den Nutzen von Cogvis insgesamt ein?
2	Support	Support	Purpose	ziel	Do you feel that Cogvis provides targeted support for your work?	Empfindest du Cogvis als zielgerichtete Unterstützung deiner Arbeit?
3	Burden	Burden	Workload	belastung	Does Cogvis place an additional burden on you?	Führt Cogvis zu einer Mehrbelastung für dich?
4	PatBenefit	Patient benefit	Patient benefit	pat_nutzen	Do you think that Cogvis benefits patients?	Denkst du, dass Cogvis den Patienten nützt?
5	SysReliabl	System Reliability	Reliability	zuverlaessig	How reliable do you consider Cogvis to be?	Wie zuverlässig schätzt du Cogvis ein?
6	FaultProne	Prone to faults	Disruptions	stoerung	How prone to faults was Cogvis?	Wie störanfällig war Cogvis?
7	MissedExit	Missed bed-exit	Misinterpretation	misswertung	How often did undetected bed exits occur?	Wie häufig kamen unerkannte Bettfluchten vor?
8	FalseAlarm	False alarm	False alarms	fehlalarm	How often did false alarms occur?	Wie häufig kamen Fehlalarme vor?
9	EaseOfUse	Ease of use	Usability	bedienung	How easy is Cogvis to use?	Wie einfach ist die Bedienung von Cogvis?
10	TrainEffort	Training effort	Learnability	neueMA	How much effort is involved in explaining Cogvis to a new member of staff?	Wie gross ist der Aufwand, einer/m neuen Mitarbeitenden Cogvis zu erklären?
11	Watched	Watched	Surveillance	beobachtet	Do you feel as though Cogvis is watching you?	Fühlst du dich durch Cogvis beobachtet?
12	Disturbed	Disturbed	Privacy	gestoert	Do you feel disturbed by Cogvis?	Fühlst du dich durch Cogvis gestört?
13	DataPriv	Data privacy	Data security	datenschutz	Do you have any concerns about data protection with Cogvis?	Hast du bei Cogvis Bedenken in Bezug auf Datenschutz?
14	MobAlerts	Mobile alerts	Mobile alerts	handy	Do you prefer to receive alerts on your mobile phone?	Bevorzugst du Alarme auf dem Handy?

Mapping of publication labels, internal variable names, and questionnaire items used in the analyses. The English questionnaire wording is presented using the Cogvis 2021 questionnaire as the reference version. Equivalent QUMEA questionnaires contained identical or only minor wording adaptations.

Table S6: Sensitivity analyses of staff acceptance of QUMEA across survey years										
**Model**	**Question-naires**	**Item responses**	**Survey year LR chi-square (df = 2)**	**Survey year *p***	**2021 vs 2024 OR (95% CI)**	**(2021 vs 2024) *p***	**2021 vs 2022 OR (95% CI)**	**(2021 vs 2022) *p***	**2022 vs 2024 OR (95% CI)**	**(2022 vs 2024) *p***
Binary GLMM, adjusted for profession	49	644	11.36	0.003	3.27 (1.19-8.96)	0.021	7.43 (2.37-23.27)	<0.001	0.44 (0.18-1.08)	0.072
Ordinal CLMM, adjusted for profession	49	644	7.96	0.019	1.92 (0.97-3.78)	0.061	3.33 (1.49-7.43)	0.003	0.58 (0.29-1.16)	.121
Binary GLMM, no profession, all questionnaires	52	671	15.40	<0.001	4.10 (1.41-11.89)	0.009	10.81 (3.45-33.87)	<0.001	0.38 (0.15-0.97)	0.042
Binary GLMM, no profession, same 49 questionnaires	49	644	14.47	<0.001	4.12 (1.43-11.83)	0.009	10.59 (3.26-34.36)	<0.001	0.39 (0.15-1.04)	0.059
Ordinal CLMM, no profession, all questionnaires	52	671	14.04	<0.001	2.67 (1.21-5.89)	0.015	5.88 (2.46-14.06)	<0.001	0.45 (0.21-0.99)	0.046
Binary GLMM, nursing staff only	44	579	14.21	<0.001	5.33 (1.72-16.51)	0.004	10.78 (3.18-36.52)	<0.001	0.49 (0.20-1.21)	.123
Ordinal CLMM, nursing staff only	44	579	8.79	0.012	2.38 (1.09-5.17)	0.029	3.97 (1.65-9.54)	0.002	0.60 (0.28-1.26)	.178
Binary GLMM, undisclosed profession = Other	52	671	13.99	<0.001	3.39 (1.23-9.34)	0.018	8.26 (2.84-24.02)	<0.001	0.41 (0.17-0.97)	0.043
Ordinal CLMM, undisclosed profession = Other	52	671	13.53	0.001	2.08 (1.02-4.23)	0.043	4.74 (2.17-10.35)	<0.001	0.44 (0.22-0.88)	0.020

CI, confidence interval; CLMM, cumulative link mixed model; df, degrees of freedom; GLMM, generalised linear mixed model; LR, likelihood ratio; OR, odds ratio.
Binary GLMM: mixed-effects logistic regression of positive (+1/+2) versus negative (−2/−1) responses. Ordinal CLMM: cumulative link mixed model (proportional odds, logit link) of the original four-point responses (−2, −1, +1, +2). All models included survey year as a fixed effect and random intercepts for questionnaire and item. Models adjusted for professional group additionally included professional group as a fixed effect. "All questionnaires" includes the three questionnaires from 2022 without information on professional group; "undisclosed profession = Other" assigns these questionnaires to other healthcare professionals; "nursing staff only" excludes other healthcare professionals. The overall effect of survey year was assessed using an LR test (df = 2). ORs with 95% CIs refer to the odds of a positive response (binary models) or of a response in a higher category (ordinal models) in the first versus the second year of each comparison. Pairwise comparisons were not adjusted for multiplicity. Responses "cannot be assessed" were treated as missing.
